# Optimization of formulation and processing conditions for the production of functional noodles containing orange‐fleshed sweet potatoes and biofortified beans

**DOI:** 10.1002/fsn3.4167

**Published:** 2024-04-12

**Authors:** Janet Natocho, Robert Mugabi, John H. Muyonga

**Affiliations:** ^1^ Department of Food Technology and Nutrition, School of Food Technology, Nutrition and Bioengineering Makerere University Kampala Uganda

**Keywords:** biofortified crops, micronutrients, nutrient‐enriched noodles, response surface methodology, sensory evaluation

## Abstract

The global demand for noodles continues to increase due to their convenience, wide appeal, and affordability. Instant noodles, in particular, are popular for their easy preparation. With annual consumption reaching 106 billion servings in 2019, there is a growing awareness of the importance of healthy food options. However, most noodle types currently available commercially are of low nutritional value. This study sought to develop a protocol for the production of functional noodles consisting of orange‐fleshed sweet potatoes (OFSP) puree and biofortified bean powder (BBP). Response surface methodology (RSM) was used to optimize product formulation and processing parameters. Reduced quartic models were found to adequately represent the relationship between dependent variables (hardness, moisture, protein, dietary fiber, iron, and zinc content) and independent variables (dough thickness, drying temperature, and drying time). *R* ^2^ values were 0.86–0.99, with a nonsignificant lack‐of‐fit (*p* < .05). Using numerical optimization, the optimal protocol for the production of functional noodles was determined to include formulation consisting of wheat 73%, OFSP 21.5%, and BBP 5.5%; dough thickness of 2.0 mm; drying temperature and time of 80.0°C and 143.4 min, respectively. These conditions yielded noodles with 5.9% moisture, 11.0 *N* hardness, 34.5% protein, 11.9% dietary fiber, 86.9 ppm (parts per million) iron, and 50.53 ppm zinc, with a desirability value of 0.82. Experimental validation demonstrated no significant difference from predicted values. Sensory evaluation rated the noodles as acceptable to consumers, with an overall acceptability of 7.8 on a 9‐point hedonic scale. These results show the potential of OFSP and BBP as ingredients for acceptable and nutrient‐rich noodles.

## Graphical abstract

This study explores optimal formulation and processing conditions to produce nutrient‐rich noodles enriched with orange‐fleshed sweet potatoes and biofortified beans. The research aims to enhance the nutritional value of noodles, offering a healthier dietary choice for consumers seeking improved nutrition.

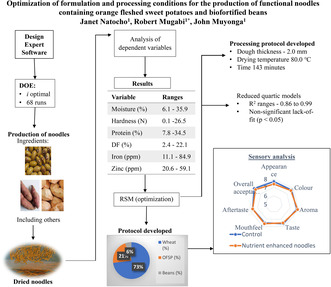

## 1 INTRODUCTION

Noodles, one of the earliest culinary innovations, are a fundamental component of dietary traditions in various areas across the globe (Akonor et al., 2017). They have become globally popular, especially due to their convenience (Adedotun et al., 2015; Sun et al., 2019), acceptable taste to different age groups, affordability, extended shelf life, and availability (Ginting & Yulifianti, 2015). The most popular basic ingredients for the production of noodles include wheat flour, water, and salt (Tiony & Irene, 2021). However, other additives can be used for purposes of enhancing the nutritional value, shelf life, texture, and eating qualities of noodles (Adejuwon et al., 2020; Siddeeg et al., 2018).

The consumption of noodles has increased considerably due to the fast‐paced lifestyle of many people (Huh et al., 2017; Idrish et al., 2014). Recent research efforts have been directed toward the improvement of the nutritional value of noodles through fortification with proteins, minerals, and vitamins (Adejuwon et al., 2020). This has created the need to manufacture a variety of noodle types based on different ingredients. Previous studies have established the possibility of using different non‐wheat crops and their processed flours in the production of noodles (Adejuwon et al., 2020; Akonor et al., 2017).

The promotion of the utilization of composite flours containing a mixture of wheat and non‐wheat sources is desirable, particularly in countries with no or low wheat production, as it enables wheat import substitution (Adejuwon et al., 2020). The focus toward wheat substitution has been placed on tuber crops, such as cassava, plantain, yam, sweet potato, and cocoyam; protein‐rich grains, such as soy, pigeon pea, and Bambara groundnut, among others; and cereal grains like rice, maize, and sorghum (Akonor et al., 2017). A number of these commodities are starchy, serving as alternative sources of the starch from wheat and some are high in dietary fiber, carotenoids, and minerals and therefore enhance the nutritional value of noodles (Adejuwon et al., 2020; Kudake et al., 2017).

Orange‐fleshed sweet potato (OFSP) and biofortified beans are among the widely produced biofortified foods in many countries. While these crops have many nutritional benefits and are versatile ingredients, there is a paucity of literature on their use in the production of noodles (Olaniran et al., 2023). OFSP is rich in energy and beta‐carotene, a vitamin A precursor (Olaniran et al., 2023). Incorporating OFSP as an ingredient in processed foods is an effective way to enhance the nutritional value of the products and alleviate vitamin A deficiency (VAD) (Low et al., 2009). Biofortified beans, on the other hand, are rich in proteins, iron, and zinc (Beebe, 2020). Processing of beans can help to increase their protein bioavailability and improve their shelf life. Biofortified beans can be processed into flour and used as an ingredient in a range of products (Nkundabombi et al., 2016).

Response surface methodology (RSM) is a statistical technique used in food product development to optimize and improve various aspects of food products, thereby improving performance and enhancing process efficiency (Yolmeh & Jafari, 2017). It can be applied in food product development to optimize the recipe of a food product by adjusting ingredient proportions and processing conditions to achieve desired sensory attributes (e.g., taste, texture, and color) or nutritional profiles while minimizing production costs (Kidane, 2021). Studies on various products like high‐amylose starch yellow alkaline noodle formula (Mojarrad & Rafe, 2018), extruded cocoyam (*Xanthosoma sagittifolium*) noodles (Sobowale et al., 2018), athletic pasta formulation (Kamali Rousta et al., 2020), novel multigrain pasta (Kamali Rousta et al., 2021), millet supplemented pasta (Gull et al., 2015), and multigrain fermented noodles (Ahmad et al., 2023), among others have been undertaken using response surface methodology.

This study sought to utilize response surface methodology to develop a protocol for the production of nutritionally enhanced noodles containing OFSP and biofortified beans as major ingredients.

## 2 MATERIALS AND METHODS

### 2.1 Selection of raw materials used in the study

The orange‐fleshed sweet potatoes used in this study were of the NASPOT 8 variety and were obtained from farmers in the Kamuli district, Uganda. NASPOT 8 was selected due to its high beta‐carotenoid content and wide availability in Uganda. Sweet potatoes used were fresh (within 48 h of harvesting), firm, with smooth skin, and uniform purple color. The beans used were of NAROBEAN 3 variety and were purchased from a market in Kampala, Uganda. The NAROBEAN 3 beans variety was selected due to its high availability in Uganda and its high iron and zinc content as compared to the other biofortified bean varieties. Refined all‐purpose wheat flour and iodized salt used in the study were procured from a retail supermarket in Kampala, Uganda.

All reagents used in the study were of analytical grade (AR), manufactured by GRIFFCHEM fine chemicals (Nairobi, Kenya) and Loba Chemie Pvt Ltd (Mumbai, India).

### 2.2 Preparation of raw materials

Orange‐fleshed sweet potatoes were washed, manually sorted to remove any diseased or infected roots, and cut into smaller pieces on a chopping board, before blending using a wonder chopper (Sokany SK‐7017–Ranzlakaz.mu, Quincaillerie ZS Hardware Store, Mauritius). Biofortified beans were sorted manually to remove any discolored, damaged, broken, and infected beans as well as foreign matter; washed and soaked in mineral water in a ratio of 1:3 for 8 h (Siddiq et al., 2011), followed by the removal of seed coat by hand and pressure cooking for 30 min. The cooked beans were then drained and dried in a hot air oven (Gallenkamp, UK) at 75°C for 6 h (Ndagire et al., 2015), left to cool at room temperature for 5 min, milled using a grinding mill (Kenwood blender BLP10), sieved using a metallic sieve (0.5 mm) and the flour was stored in polyethylene (PE) zipper bags until required for use. Wheat flour was also sieved and packaged the same way as bean powder.

### 2.3 Design of experiment

The I optimal of the Design‐Expert®12 statistical software (Stat‐Ease, Inc., Minneapolis, USA) was used to generate the experimental runs for this study (Ataei Nukabadi et al., 2021; Vaka et al., 2020). The purpose was to develop an optimal formulation and process conditions for the production of noodles enriched with OFSP puree and BBP. The independent variables included the content of wheat flour, orange‐fleshed sweet potatoes, and biofortified beans, drying temperature and drying time (process parameters) as continuous variables, and dough thickness under processing parameters as discrete variables. The upper and lower limits for ingredients and processing parameters (Table 1) were decided based on results from preliminary experiments. A total of 68 experimental runs were generated (Table 2). The six response variables selected were moisture content, hardness, protein, dietary fiber, iron, and zinc content. Interaction between any two independent variables was visualized by surface plots in which the third variable was held constant.

**TABLE 1:** The components used to study their effect on different response variables.

Factor	Symbol	Range and levels	
		–1	+1
Wheat flour	$X_{1}$	60	90
Orange‐fleshed sweet potato	$X_{2}$	1*5*	2*5*
Biofortified beans	$X_{3}$	5	10
Dough thickness	$X_{4}$	2	4
Drying temperature	$X_{5}$	80	100
Drying time	$X_{6}$	60	150

**TABLE 2:** Experimental runs generated for optimization of production of functional noodles.

Run	A: Wheat (g)	B: Ofsp (g)	C: Beans (g)	D: Thickness (mm)	E: Temperature (°C)	F: Time (min)	Moisture (%)	Hardness (*N*)	Protein (%)	Dietary fiber (%)	Iron (ppm)	Zinc (ppm)
1	75.00	20.00	7.50	3.00	90.00	105.00	14.46	12.41	23.00	5.23	34.62	53.41
2	60.00	15.00	5.00	4.00	89.50	60.00	27.19	0.12	22.31	6.79	35.70	54.10
3	60.00	18.50	10.00	2.00	100.00	150.00	6.14	7.80	18.78	2.37	65.27	45.51
4	90.00	15.00	6.98	2.00	91.10	150.00	6.80	13.17	20.05	7.33	29.76	45.10
5	90.00	15.00	5.00	4.00	80.00	101.85	22.80	3.34	14.81	5.87	88.20	51.78
6	72.00	22.30	10.00	4.00	86.07	127.95	13.75	18.09	22.46	8.53	46.44	51.66
7	67.50	17.75	5.00	2.00	80.00	69.00	25.38	0.12	20.27	9.05	71.09	59.05
8	60.00	25.00	10.00	3.00	80.00	100.50	18.72	0.12	22.77	13.85	60.62	49.82
9	60.00	15.00	5.00	2.00	100.00	109.05	8.78	16.00	23.30	3.49	34.94	49.44
10	90.00	15.00	10.00	4.00	90.20	78.00	18.57	4.19	25.98	4.48	93.54	56.63
11	80.85	25.00	7.33	4.00	80.00	60.00	29.04	0.12	34.48	7.73	73.01	52.56
12	90.00	25.00	8.38	4.00	80.00	150.00	14.42	12.68	17.02	14.08	53.34	52.61
13	90.00	15.00	10.00	2.00	100.00	101.40	10.06	13.91	17.99	10.31	11.10	20.60
14	90.00	19.85	5.00	3.00	88.50	150.00	11.09	17.54	17.67	11.78	84.96	41.57
15	90.00	19.85	5.00	3.00	88.50	150.00	10.83	15.70	12.53	11.11	73.80	37.10
16	60.00	15.00	7.25	3.00	80.00	103.65	14.43	4.25	25.61	6.16	49.68	54.58
17	72.15	15.00	5.00	2.00	80.00	150.00	14.36	9.21	21.04	5.25	57.72	55.26
18	60.00	15.00	9.88	4.00	89.50	150.00	12.03	14.57	22.76	22.05	64.64	28.85
19	90.00	25.00	10.00	3.00	91.40	60.00	26.58	0.12	18.26	6.51	53.82	55.10
20	60.00	25.00	5.00	3.00	80.00	60.00	35.85	0.12	22.46	8.23	72.09	53.57
21	75.75	20.80	5.00	2.00	95.00	60.00	13.05	9.21	22.94	5.27	41.33	54.21
22	75.00	20.00	7.50	3.00	90.00	105.00	14.18	12.43	24.69	3.98	50.52	51.51
23	75.00	20.00	7.50	3.00	90.00	105.00	13.38	11.80	25.62	4.61	42.63	52.50
24	75.00	20.00	7.50	3.00	90.00	105.00	14.76	9.38	25.15	5.02	42.57	52.46
25	75.92	25.00	10.00	2.00	92.00	150.00	9.11	5.48	22.64	3.68	56.46	49.79
26	60.00	15.00	10.00	2.00	85.20	60.00	28.48	0.11	2.96	7.93	44.45	50.47
27	75.00	20.00	7.50	3.00	90.00	105.00	14.19	11.20	25.03	4.18	48.32	51.98
28	90.00	20.65	5.13	3.00	84.60	60.00	22.59	2.38	8.43	7.67	44.45	55.48
29	90.00	25.00	10.00	3.00	91.40	60.00	26.86	0.12	19.11	8.49	57.00	52.75
30	69.90	19.20	10.00	4.00	80.00	60.00	28.42	0.12	20.50	7.66	54.48	24.72
31	90.00	25.00	5.00	2.00	80.00	105.00	16.26	5.01	24.80	5.84	67.35	57.27
32	75.75	15.00	5.75	4.00	100.00	150.00	8.89	25.77	21.61	1.38	69.09	47.61
33	75.00	20.00	7.50	3.00	90.00	105.00	14.31	9.60	23.08	4.98	46.13	53.21
34	75.00	20.00	7.50	3.00	90.00	105.00	13.98	12.22	25.98	3.90	39.88	52.81
35	90.00	24.00	7.85	2.00	100.00	60.00	13.49	7.62	9.89	3.69	56.24	50.09
36	79.50	21.25	10.00	4.00	100.00	71.17	15.34	5.25	21.06	10.47	53.72	50.25
37	76.37	15.02	9.55	4.00	100.00	121.00	10.93	26.45	23.33	3.59	45.17	47.15
38	75.00	20.00	7.50	3.00	90.00	105.00	15.17	12.63	24.12	4.38	45.79	51.78
39	60.00	25.00	8.13	2.00	100.00	60.00	12.67	7.09	18.22	5.24	63.41	44.81
40	90.00	20.75	10.00	2.00	80.00	100.50	18.39	0.12	18.55	11.84	61.53	54.70
41	90.00	18.40	5.00	2.00	100.00	110.85	9.96	12.60	27.60	3.40	48.51	53.51
42	81.90	15.00	10.00	3.00	80.00	150.00	13.59	12.22	21.51	9.44	56.73	57.34
43	60.00	15.00	7.25	3.00	80.00	103.65	14.59	5.05	21.49	8.31	55.47	52.16
44	90.00	25.00	5.00	4.00	100.00	64.05	17.00	7.33	22.31	14.29	42.66	45.89
45	90.00	21.05	10.00	4.00	100.00	150.00	10.85	21.73	21.75	1.15	65.36	29.17
46	60.00	25.00	7.32	2.00	80.00	150.00	13.69	8.87	23.23	4.44	70.31	51.45
47	89.25	25.00	7.79	4.00	93.50	109.02	20.57	0.12	10.33	4.29	41.39	48.95
48	72.15	15.00	10.00	3.00	100.00	60.00	14.98	7.17	24.59	11.33	43.97	47.96
49	60.00	22.00	7.15	4.00	84.00	88.75	17.57	3.69	7.82	6.18	48.29	51.90
50	86.55	17.03	9.68	3.00	80.00	60.00	28.55	0.12	18.22	6.39	54.51	52.43
51	75.92	25.00	10.00	2.00	92.00	150.00	8.92	6.88	19.88	2.85	50.28	45.30
52	60.00	18.75	5.00	4.00	95.90	115.80	9.92	22.19	25.58	9.28	37.26	54.22
53	60.00	19.50	7.38	4.00	100.00	60.00	23.00	0.12	19.01	6.05	43.20	48.24
54	74.70	25.00	6.50	3.00	100.00	78.90	11.82	12.69	20.02	6.29	70.13	49.60
55	60.00	25.00	5.00	3.00	100.00	150.00	9.89	21.93	23.86	2.94	39.53	48.46
56	90.00	25.00	6.75	2.00	100.00	140.55	6.95	7.85	22.68	4.69	52.59	23.40
57	90.00	15.00	5.00	3.00	100.00	60.00	13.34	7.60	19.37	4.60	50.09	49.51
58	60.00	23.05	8.54	3.00	93.10	150.00	10.37	21.57	20.93	3.50	43.28	47.12
59	75.00	20.00	7.50	3.00	90.00	105.00	13.52	13.00	25.36	4.59	46.63	52.69
60	81.90	15.00	10.00	3.00	80.00	150.00	14.30	12.40	22.89	10.11	49.80	51.77
61	90.00	20.75	10.00	2.00	80.00	100.50	19.83	2.73	22.04	4.22	46.20	50.43
62	90.00	15.00	10.00	4.00	90.20	78.00	17.88	4.02	28.46	5.43	72.70	54.80
63	75.75	15.00	5.75	4.00	100.00	150.00	6.60	11.98	23.65	3.92	53.24	49.50
64	72.00	25.00	5.00	4.00	90.60	118.50	15.97	7.06	17.58	8.08	49.20	49.59
65	60.00	20.29	5.00	4.00	80.00	150.00	19.28	0.13	15.53	4.85	54.77	52.40
66	60.00	15.00	10.00	2.00	85.20	60.00	29.27	0.13	2.84	7.74	40.73	56.28
67	60.00	25.00	10.00	4.00	100.00	106.80	11.02	7.38	22.86	3.13	52.62	32.09
68	90.00	15.00	6.88	2.00	80.00	60.00	19.88	0.12	19.38	7.45	58.20	53.35

*Note*: Results are means obtained from triplicate individual values of response variables (moisture, hardness, protein, dietary fiber, iron, and zinc).

The empirical model (Equation 1) given below was used to represent the relationships between the independent variables and the response variables.

(1) $$Y=B_{0}+\sum B_{i}X_{i}+\sum B_{\mathrm{ij}}X_{i}X_{j}+\sum B_{\mathrm{ii}}{X_{i}}^{2},$$

where *Y* indicates the response output (moisture, hardness, protein, dietary fiber, iron, and zinc content), *B*_*o*_ is the independent constant, *B*_*i*_ is the linear/individual effect, *B*_*ij*_ is the interaction effect (*i* = *A*, *B*, *C*, *D*, *E*, and *F* and *j* = *A*, *B*, *C*, *D*, *E*, and *F*), *B*_*ii*_ is the squared effect, and *X*_*i*_ is the independent experimental components (*i* = *A*, *B*, *C*, *D*, *E*, and *F*).

### 2.4 Production of noodles

Noodles were produced according to the method described by Adejuwon et al. (2020) with modifications following the experimental conditions shown in Table 2. The ingredients were weighed and mixed using a mixer (Sokany Mini Wonder Chopper SK‐7005, Zhejiang Province, China). The dough was kneaded by hand and then put into a pasta maker (Marcato Design Atlas 150 Pasta Machine, Campodarsego, Italy). Thickness was set as per the experimental runs generated. The noodles were steamed for 10 min and then dried in a Gallenkamp hot air oven (Gallenkamp, Cambridge, UK). After drying, they were air‐cooled for 10 min and packaged in polyethylene (PE) zipper bags until required for laboratory analysis.

### 2.5 Analytical methods

#### 2.5.1 Chemical analysis of functional noodles

The chemical analysis comprised moisture content, protein, and dietary fiber. The moisture content was determined using the official method of 934.01 (AOAC, 2016), protein (*N* × 6.25) was determined using the official method of 979.09 (AOAC, 2000), and crude fiber was determined using the official method of 978.10 (AOAC, 2005).

#### 2.5.2 Iron and zinc content determination

Atomic absorption spectroscopy (Agilent 247FS) according to the Association of Official Analytical Chemists (AOAC) (2010) was used to determine the concentration of iron and zinc in samples (Paul et al., 2016; Paul et al., 2017). One gram of sample was used in the experiment. The wavelength for iron and zinc was set at 248.3 nm and 213.9 nm, respectively. Results were expressed as mg/100 g sample (Tiony & Irene, 2021).

#### 2.5.3 Determination of hardness

The hardness of dried noodles strands was measured using a Texture Analyser (TA‐XTplus, Stable Micro Systems, Godalming, UK), as described by Nansereko et al. (2022) with slight modifications. The hardness of each single strand of dry noodles of about 10 cm long was determined with a 3‐point bending rig with TA‐TPB from a general probe kit; 5 kg load cell and the pretest, test, and posttest speeds were 1.5, 2, and 10 mm/s, respectively, with a 3 mm compression distance. The noodle strands were placed between the TA‐TPB fixture and the fixture base. The upper rig was lowered toward the sample. It eventually created pressure with the lower rigs and bent the noodle strand until it broke. The maximum force (*N*) during compression was recorded. Results were obtained in quadruplicate.

### 2.6 Optimization and validation

Multiresponse numerical optimization, based on the desirability function, was undertaken using the Design‐Expert Version 12 software (Akonor et al., 2021). The response variables measured (moisture content, hardness, protein, dietary fiber, iron, and zinc content) were individually expressed as a function of the independent variable, as shown in Equations (2), (3), (4), (5), (6), (7). The significance of the models was determined using model p‐value and lack‐of‐fit. During optimization, moisture content was minimized, while hardness, protein, dietary fiber, iron, and zinc were maximized. Noodles were processed using the predicted optimized conditions, and experimental values of the response variables were determined and compared with the theoretical values using the *t*‐test (*p* < .05) in Microsoft Excel 2021 to establish the validity of predictions.

### 2.7 Sensory evaluation

Fifty semi‐trained panelists assessed coded samples of noodles. Each panelist received two samples of noodles prepared following the procedure described by Food Network Kitchen (2022), one sample was prepared using wheat as the sole flour (control) and the other sample consisted of noodles produced using optimal conditions determined in Section 2.6 above. The sensory acceptance (appearance, color, aroma, taste, flavor, mouth feel, and overall acceptability) of the noodles was assessed using a 9‐point hedonic scale, where 1 = dislike extremely and 9 = like extremely (Akajiaku et al., 2017).

### 2.8 Ethical considerations

Research protocols were approved by the Research Committee of the School of Food Technology, Nutrition and Bioengineering, Makerere University, and all the participants provided informed consent.

### 2.9 Statistical analysis

Design‐Expert Version 12 software was used to analyze the data. For each response variable (moisture, hardness, protein, dietary fiber, iron, and zinc content) different models and their suitability were evaluated. The significant terms in the generated models were determined by analysis of variance (ANOVA) using the Statistical Software Program (SPSS) at a 5% probability level. The model's lack‐of‐fit, coefficient of determination (*R* ^2^), and adjusted *R* ^2^ were used to test the adequacy of the developed models. Good mathematical fitting models with a large *R* ^2^ (larger than 80%) and adjusted *R* ^2^ were selected.

## 3 RESULTS AND DISCUSSION

### 3.1 Model fitting

The models for all response variables (moisture content, hardness, protein, dietary fiber, iron, and zinc) were significant (*p* < .05) and had a nonsignificant (*p* > .05) lack‐of‐fit. This showed that the models developed adequately explained the data. The reduced quartic models (Equations (2), (3), (4), (5), (6), (7)) show the best fit for the relation between the independent variables and the different response variables (moisture content, hardness, protein, dietary fiber, iron, and zinc).

(2) $$\begin{array}{c} \text{Moisture content}\;\left(\%\right)=-1681.5+\left(28.5X_{1}\right)\\ +\left(147.6X_{2}\right)+\left(135.7X_{3}\right)+\left(-343.3X_{4}\right)+\left(30.6X_{5}\right)\\ +\left(-5.8X_{6}\right)+\left(-1.7X_{1}X_{2}\right)+\left(4.9X_{1}X_{4}\right)+\left(0.01X_{1}X_{6}\right)\\ +\left(-17.0X_{2}X_{3}\right)+\left(11.9X_{2}X_{4}\right)+\left(-2.2X_{2}X_{5}\right)\\ +\left(0.1X_{2}X_{6}\right)+\left(40.5X_{3}X_{4}\right)+\left(1.6X_{3}X_{6}\right)+\left(1.5X_{4}X_{5}\right)\\ +\left(0.1X_{4}X_{6}\right)+\left(-0.02X_{5}X_{6}\right)+\left(-0.4{X_{2}}^{2}\right)\\ +\left(13.5\;{X_{3}}^{2}\right)+\left(0.02{X_{6}}^{2}\right), \end{array}$$

(3) $$\begin{array}{c} \text{Protein content}\;\left(\%\right)=-1849.0+\left(9.7X_{1}\right)+\left(85.1X_{2}\right)\\ +\left(-60.6X_{3}\right)+\left(318.8X_{4}\right)+\left(19.1X_{5}\right)+\left(27.3X_{6}\right)\\ +\left(1.1X_{1}X_{3}\right)+\left(-0.6X_{1}X_{4}\right)+\left(-0.1X_{1}X_{5}\right)+\left(-0.3X_{1}X_{6}\right)\\ +\left(6.5X_{2}X_{3}\right)+\left(-9.5X_{2}X_{4}\right)+\left(-1.2X_{2}X_{5}\right)+\left(-1.4X_{2}X_{6}\right)\\ +\left(-40.9X_{3}X_{4}\right)+\left(1.6X_{3}X_{5}\right)+\left(0.6X_{4}X_{6}\right)+\left(-0.02{X_{1}}^{2}\right)\\ +\left(-6.9{X_{3}}^{2}\right)+\left(2.8{X_{4}}^{2}\right), \end{array}$$

(4) $$\begin{array}{c} \text{Hardness}\;\left(N\right)=-1871.7+\left(24.4X_{1}\right)+\left(-2.7X_{2}\right)\\ +\left(153.4X_{3}\right)+\left(23.7X_{4}\right)+\left(28.1X_{5}\right)+\left(7.3X_{6}\right)+\left(0.2X_{1}X_{4}\right)\\ +\left(0.4X_{1}X_{5}\right)+\left(-0.1X_{1}X_{6}\right)+\left(-3.7X_{2}X_{3}\right)+\left(-12.3X_{3}X_{4}\right)\\ +\left(-0.1X_{3}X_{5}\right)+\left(-1.1X_{3}X_{6}\right)+\left(-0.1{X_{2}}^{2}\right), \end{array}$$

(5) $$\begin{array}{c} \text{Dietary fibre}\;\left(\%\right)=337.2+\left(-4.6X_{1}\right)+\left(-11.5X_{2}\right)\\ +\left(-31.6X_{3}\right)+\left(-48.7X_{4}\right)+\left(1.6X_{5}\right)+\left(6.8X_{6}\right)+\left(0.1X_{1}X_{2}\right)\\ +\left(0.6X_{1}X_{4}\right)+\left(0.1X_{1}X_{6}\right)+\left(-0.01X_{2}X_{4}\right)+\left(-0.1X_{2}X_{5}\right)\\ +\left(0.01X_{5}X_{6}\right)+\left(-0.5{X_{3}}^{2}\right)+\left(-1.2{X_{4}}^{2}\right), \end{array}$$

(6) $$\begin{array}{c} \text{Iron}\;\left(\mathrm{ppm}\right)=+2618.5+\left(-35.5X_{1}\right)+\left(-77.8X_{2}\right)\\ +\left(-158.2X_{3}\right)+\left(581.8X_{4}\right)+\left(-54.1X_{5}\right)+\left(0.4X_{6}\right)+\left(1.3X_{1}X_{3}\right)\\ +\left(-7.6X_{1}X_{4}\right)+\left(-34.5X_{2}X_{4}\right)+\left(5.4X_{3}X_{5}\right)+\left(0.2X_{3}X_{6}\right)+\left(0.2{X_{5}}^{2}\right), \end{array}$$

(7) $$\begin{array}{c} \text{Zinc}\;\left(\mathrm{ppm}\right)=-1848.2+\left(12.3X_{1}\right)+\left(-0.67X_{2}\right)+\left(1183.5X_{3}\right)\\ +\left(-17.8X_{4}\right)+\left(-8.8X_{5}\right)+\left(-5.8X_{6}\right)+\left(0.1X_{1}X_{3}\right)+\left(-1.1X_{1}X_{4}\right)\\ +\left(0.1X_{1}X_{5}\right)+\left(-0.3X_{1}X_{6}\right)+\left(-1.1X_{2}X_{3}\right)+\left(-0.1X_{2}X_{5}\right)\\ +\left(-12.6X_{3}X_{4}\right)+\left(0.9X_{3}X_{5}\right)+\left(-0.04X_{3}X_{6}\right)+\left(0.9X_{4}X_{5}\right)\\ +\left(0.9X_{4}X_{6}\right)+\left(0.2X_{5}X_{6}\right)+\left(-255.8{X_{3}}^{2}\right), \end{array}$$

where $X_{1}$, $X_{2}$, $X_{3}$, $X_{4}$, $X_{5}$, and $X_{6}$ are wheat flour content, orange‐fleshed sweet potatoes content, biofortified beans content, thickness, temperature, and drying time, respectively, for the processed‐enriched noodles.

### 3.2 Effect of ingredients and processing conditions on individual response variables

#### 3.2.1 Moisture content

**FIGURE 1:**
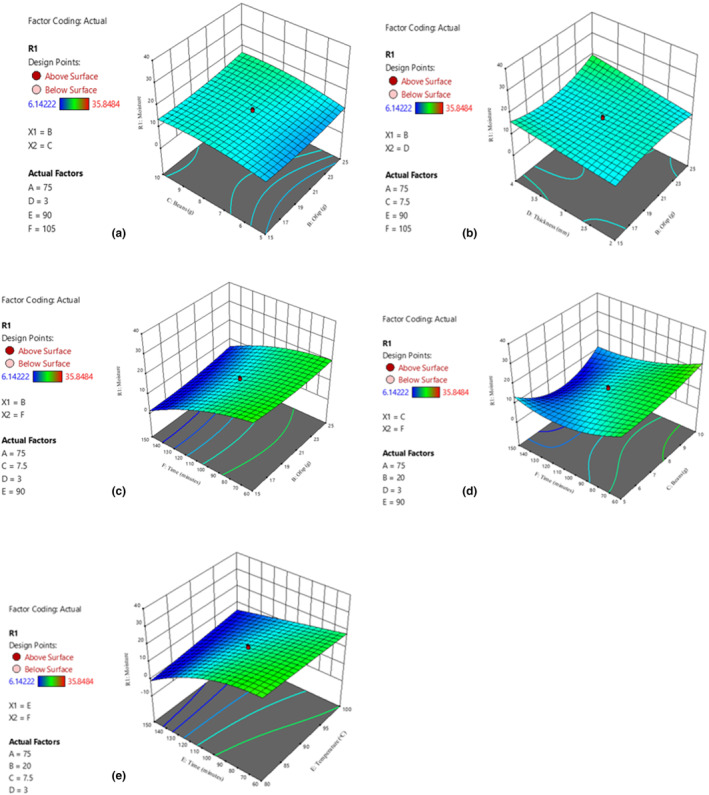
Effect of interactions on moisture content. (a) OFSP and beans; (b) OFSP and dough thickness; (c) OFSP and drying time; (d) beans and drying time; (e) dough thickness and drying temperature.

The moisture content for the nutritionally enhanced noodles produced ranged between 6.1% and 35.9%, as shown in Table 2. The highest moisture content was obtained with the combination of 60 g wheat, 25 g OFSP, 5 g beans, and a 3 mm thick dough dried at 80°C for 60 min (sample 20 in Table 2). While the lowest moisture content was observed in sample 3 (wheat – 60 g, OFSP – 18.5 g, beans – 10 g, thickness – 2 mm, drying temperature – 100°C, and drying time – 150 min). Figure 1a–e show the relationship between different independent variables (wheat, OFSP, beans, dough thickness, drying temperature, and drying time) and moisture content. The surface plots exhibit the dependence of moisture content on wheat flour, OFSP, and biofortified beans. The thickness did not have a significant effect on moisture content (*p* = .239). Both linear and quadratic terms of temperature and time had significant effect on moisture content (*p* < .05). The experimental and theoretical results were in close agreement, as indicated by the proximity between *R* ^2^ (0.99) and adjusted *R* ^2^ (0.99) values (Table 3) as the difference is less than 0.2, which indicated that the model predicted 99% of the response variable results. The moisture content equation showing the effect of independent variables is given in Equation 2.

**TABLE 3:** Statistical evaluation of response variables.

Fit statistics	Response variable					
	Moisture (%)	Hardness (*N*)	Protein (%)	Dietary fiber (%)	Iron (ppm)	Zinc (ppm)
Model *p*‐value	<0.0001	<0.0001	<0.0001	<0.0001	<0.0001	<0.0001
Lack‐of‐fit‐*p*‐value	0.12	0.14	0.13	0.19	0.27	0.12
*R* ^2^	0.99	0.90	0.95	0.90	0.86	0.96
Adjusted *R* ^2^	0.99	0.81	0.88	0.77	0.72	0.91

*Note*: Significance was considered at *p* < .05.

Processing conditions (drying temperature, thickness, and drying time) had a significant effect on the moisture content of the nutritionally enhanced noodles. It was observed that the higher the drying temperature and drying time, the lower the moisture content of the final enriched noodle. This is in agreement with results by Hakoda et al. (2006). These results can be attributed to the higher drying temperatures and prolonged drying period, which may have led to increased evaporation of water molecules from the material being dried (Asgar et al., 2022). The moisture content of the noodles increased with increase in bean flour content (Figure 4g). This may be attributed to the high water absorption capacity associated with the increased total protein and pentosan content (Bojňanská et al., 2021; Hoxha et al., 2020). Regression analysis showed both linear and quadratic terms of biofortified bean flour significantly (*p* < .05) affected the moisture content of the noodles. Also, drying time for noodles decreases with decrease in thickness. Moisture content is used as an indicator of food quality as it has a direct impact on the sensory and physical properties of food products. It is also important to know that a low moisture content indicates better stability during storage (Akonor et al., 2021; González et al., 2021). Instant noodles generally have low moisture content because their production entails dehydration, either by frying in oil or by drying. The maximum moisture permitted according to regulatory standards is 10.0% and 14.0%, respectively, for fried and non‐fried noodles (CAC, 2006).

#### 3.2.2 Protein content

**FIGURE 2:**
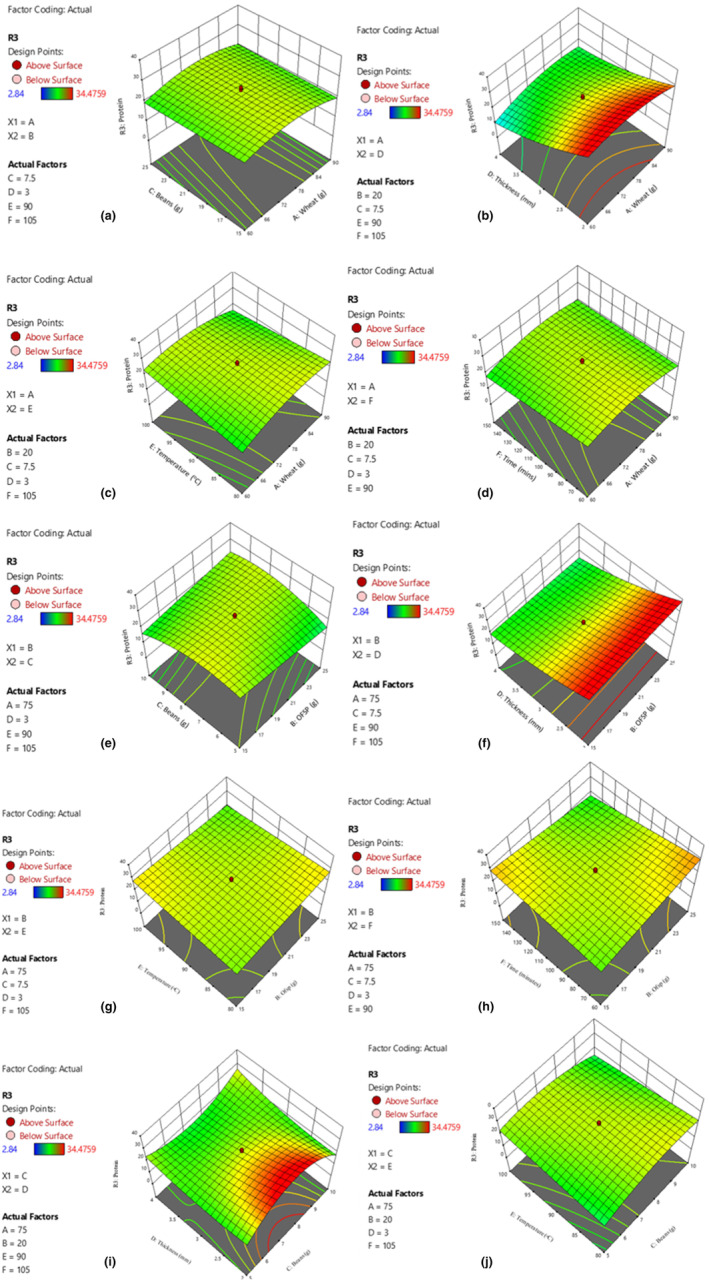
Effect of interactions on protein content. Wheat and beans (a); and wheat and dough thickness (b); wheat and drying temperature (c); and wheat and drying time (d); OFSP and beans (e); and OFSP and dough thickness (f); OFSP and drying temperature (g); and OFSP and drying time (h); beans and thickness (i); and beans and drying temperature (j).

The protein content of dried nutrient‐enriched noodles ranged between 7.8% and 34.5% (Table 2). Figure 2a–j show the relationship between independent variables (wheat, OFSP, beans, dough thickness, drying temperature, and drying time) and the protein content of the final product. The experimental and theoretical results were in close agreement, as indicated by the proximity between *R* ^2^ (0.95) and adjusted *R* ^2^ (0.88) values (Table 3) as the difference is less than 0.2. The surface plots show the dependence of protein content on biofortified beans, wheat flour, and OFSP. The relationship between the independent variables and response variable–protein content is shown in Equation 3. The linear terms of wheat, OFSP, and beans had no significant effect on protein content (*p* > .05). Whereas the quadratic terms of wheat and beans had a significant effect on protein content (*p* < .05).

Approximately 90% of the noodle samples had protein content exceeding 15% (Table 2). A study by Park and Baik (2004) reported that noodles from wheat flours with high protein content (>13.6%) exhibited low‐fat absorption and firmer and more elastic texture. The high protein content was attributed to the high biofortified bean flour content (Figure 2a,e). Biofortified beans have the potential to alleviate micronutrient malnutrition as they are rich in quality protein, fiber, and micronutrients such as iron and zinc (Saloko et al., 2020). Biofortified beans, with their inherently higher protein content, can significantly affect the protein content of noodles when used as ingredients (Beebe, 2020; Mahmoud et al., 2012). Because the amino acid profile of beans is complementary to that of wheat, noodles containing the two ingredients exhibit a superior essential amino acids profile, hence higher protein quality. Also, the protein content of the beans is retained well during processing, especially when processed into flour or incorporated into noodle dough (Audu & Aremu, 2011).

#### 3.2.3 Hardness

**FIGURE 3:**
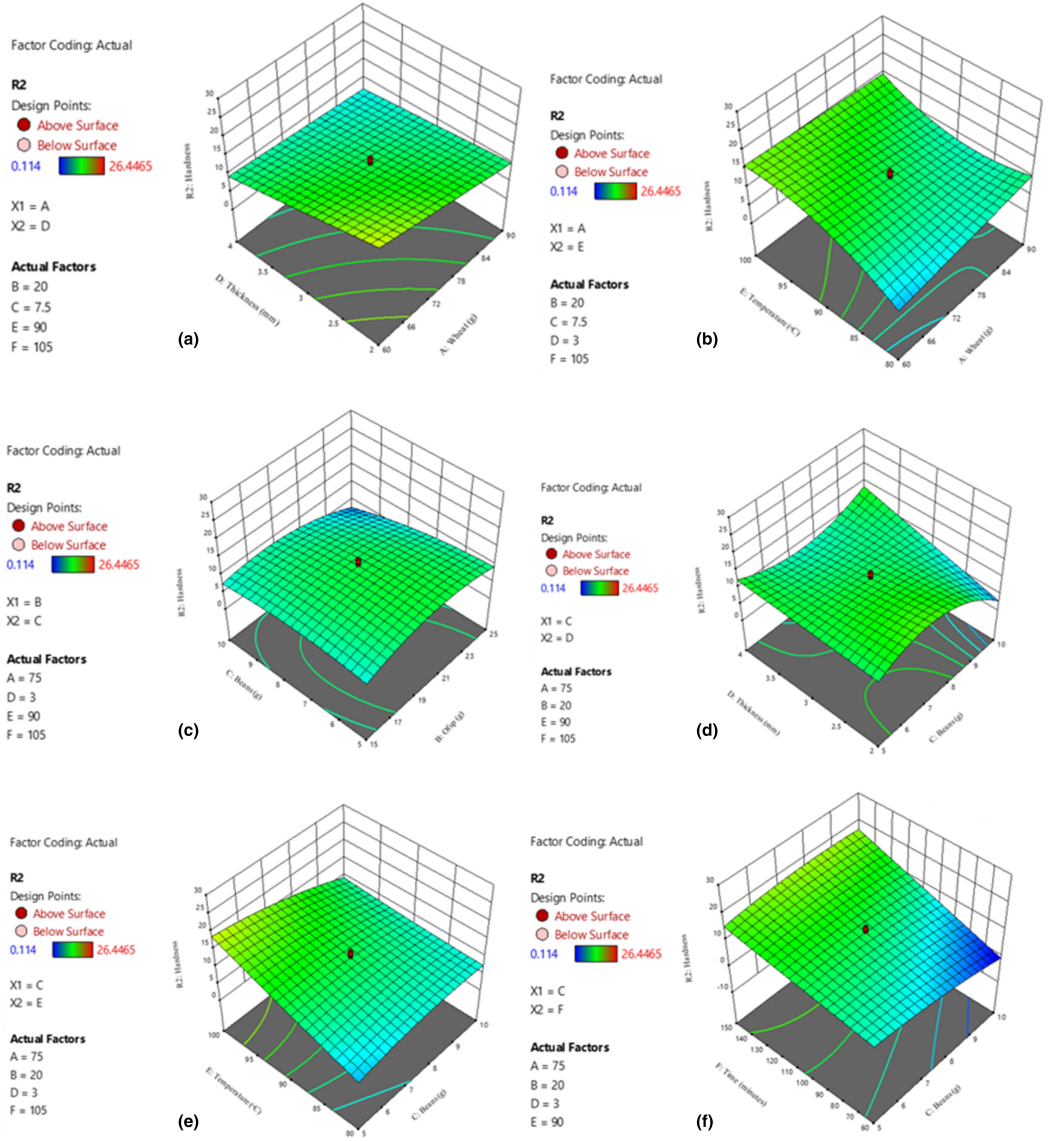
Effect of interactions on hardness. Wheat and dough thickness (a); and wheat and drying temperature (b); OFSP and beans (c); and beans and dough thickness (d); beans and drying temperature (e); and beans and drying time (f).

The hardness of dried nutrient‐enriched noodles ranged between 0.1 and 26.5 (*N*) (Table 2). Figure 3a–f show the relationship between independent variables (wheat, OFSP, beans, dough thickness, drying temperature, and drying time) and hardness. The experimental and theoretical results were in close agreement, as indicated by the proximity between *R* ^2^ (0.90) and adjusted *R* ^2^ (0.81) values (Table 3) with a difference of less than 0.2. The relationship between hardness and independent variables (ingredients and processing parameters) is given in Equation 4. The linear terms for wheat, beans, thickness, drying temperature, and drying time had a significant effect on hardness (*p* < .05). On the other hand, the quadratic term for OFSP had a significant effect on hardness (*p* < .05).

Texture, in terms of hardness, is influenced by several factors such as raw materials used and the temperature/time used during the production process (Al‐Baarri et al., 2021). Wheat flour is a primary source of gluten, which contributes to the structure and hardness of noodles. Gluten is a protein found in wheat flour that plays a crucial role in forming the protein network responsible for the firm texture of noodles (Asri et al., 2021). Grated OFSP and biofortified beans introduce additional moisture and starch content. Substituting a portion of wheat flour with OFSP reduces the overall gluten content, potentially leading to softer noodles (González et al., 2021; Meng et al., 2022).

High drying temperature and longer drying time are associated with an increase in the amount of water that evaporates from the noodles (Asgar et al., 2022), increasing the hardness of the products. Ingredients like beans increase the protein content of noodles. The proteins interact with other components during dough development of noodles, which later affects the hardness of the dried noodles (Suriya et al., 2017). Nutrient‐enriched noodles were produced by partially substituting wheat flour with orange‐fleshed sweet potatoes and beans and this increased the protein content of the final product.

#### 3.2.4 Dietary fiber

**FIGURE 4:**
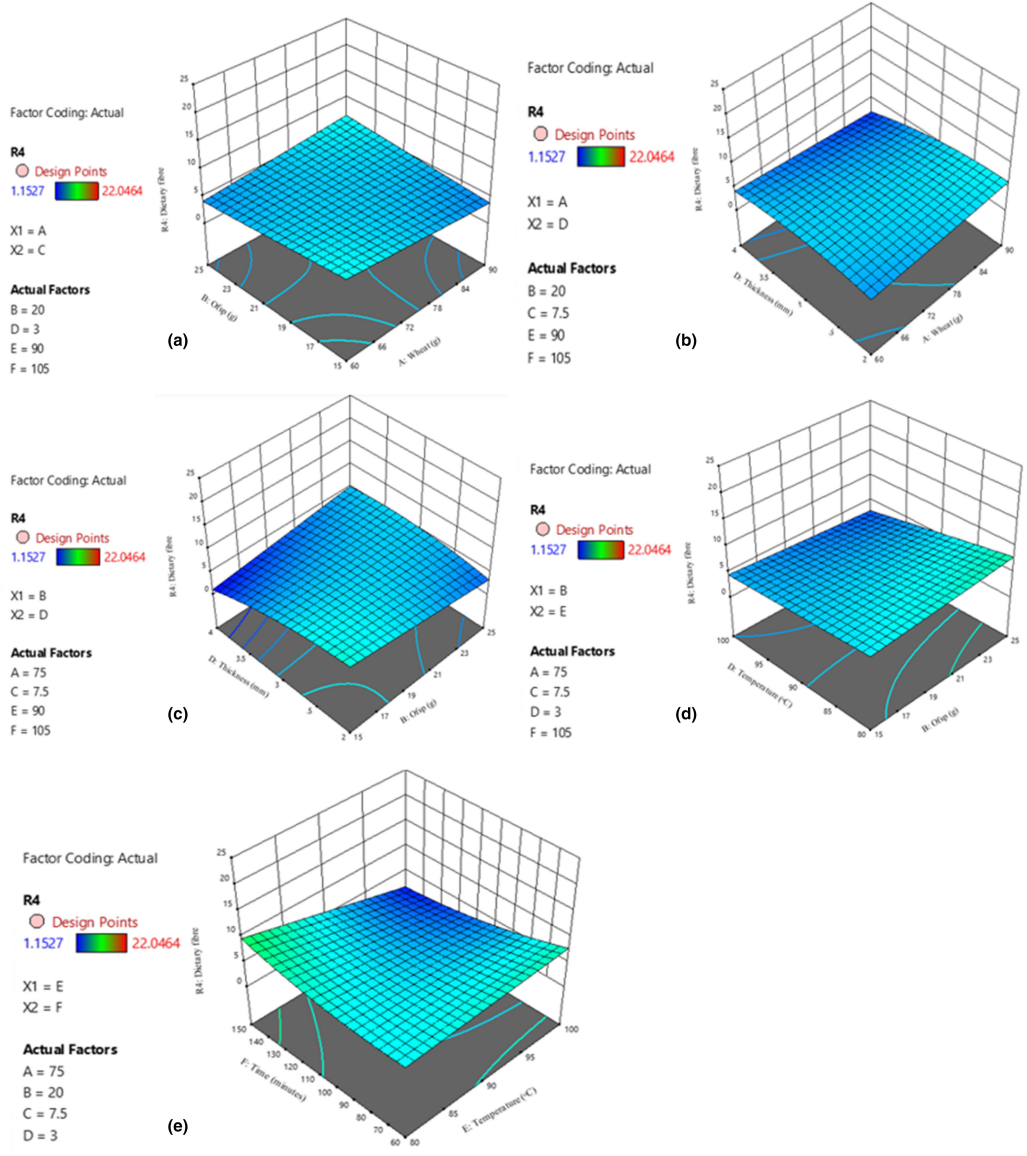
Effect of interactions on dietary fiber. Wheat and OFSP (a); and wheat and dough thickness (b); OFSP and dough thickness (c); and OFSP and drying temperature (d); drying temperature and drying time (e).

The dietary fiber of the nutrient‐enriched noodles ranged between 2.4% and 22.1% (Table 2). Figure 4a–e show the relationship between independent variables (wheat, OFSP, beans, dough thickness, drying temperature, and drying time) and dietary fiber. The experimental and theoretical results were in close agreement, as indicated by the proximity between *R* ^2^ (0.90) and adjusted *R* ^2^ (0.77) values (Table 3). The relationship between dietary fiber content and the protocol (ingredients and processing parameters) used to make the noodles is given in Equation 5. The linear and quadratic terms for wheat and OFSP did not have a significant effect on dietary fiber (*p* > .05). The linear and quadratic terms for beans, drying temperature, and drying time had significant effects on dietary fiber (*p* < .05). An increase in drying temperature concomitant with an increase in OFSP content led to a decrease in the dietary fiber content of the final product (Figure 4d). This observation can be explained by the effect that processing conditions have on the nutritional composition of orange‐fleshed sweet potatoes. A study on purple‐fleshed sweet potatoes showed that drying temperature had a significant effect on the functional properties of the final product (Vidal et al., 2022). Furthermore, Ie and Je (2018) noted that incorporating OFSP flour into mixes was linked to modifications in the products’ moisture, ash, fiber, and β‐carotene levels, hence, suggesting a possible decrease in fiber content as OFSP content increased. Also, Okoronkwo et al. (2019) suggested that the high ash level of OFSP flour, which is an indicator of a higher mineral content due to biofortification, may influence the product's total dietary fiber content.

Dietary fiber is an essential component of a healthy diet, and its presence in nutritionally enhanced noodles has an added advantage as it is essential for digestive health (Barber et al., 2020). The results of this study show that the higher the raw OFSP and bean powder used in the production of noodles, the higher the dietary fiber content of the final product. Biofortified bean powder and grated raw orange‐fleshed sweet potatoes are naturally rich in dietary fiber (Adoko et al., 2021). Beans are known for their high fiber content, including both soluble and insoluble fiber (Brick et al., 2022), while orange‐fleshed sweet potatoes also contain dietary fiber, particularly soluble fiber (Neela & Fanta, 2019), which contributes to their nutritional value. Partial substitution of wheat flour, with these ingredients, in the production of noodles effectively results in noodles with higher dietary fiber content (Adoko et al., 2021). It is also noted that the dietary fiber in these ingredients is generally stable and resistant to degradation during processing (Elleuch et al., 2011). This can be beneficial for people seeking to increase their fiber intake, as a high‐fiber diet has been linked to numerous health benefits, including improved digestive health (Malavi et al., 2022), lower risk of heart disease and strdetke, and lower risk of certain types of cancer (Pakhare et al., 2018).

#### 3.2.5 Iron and zinc content

**FIGURE 5:**
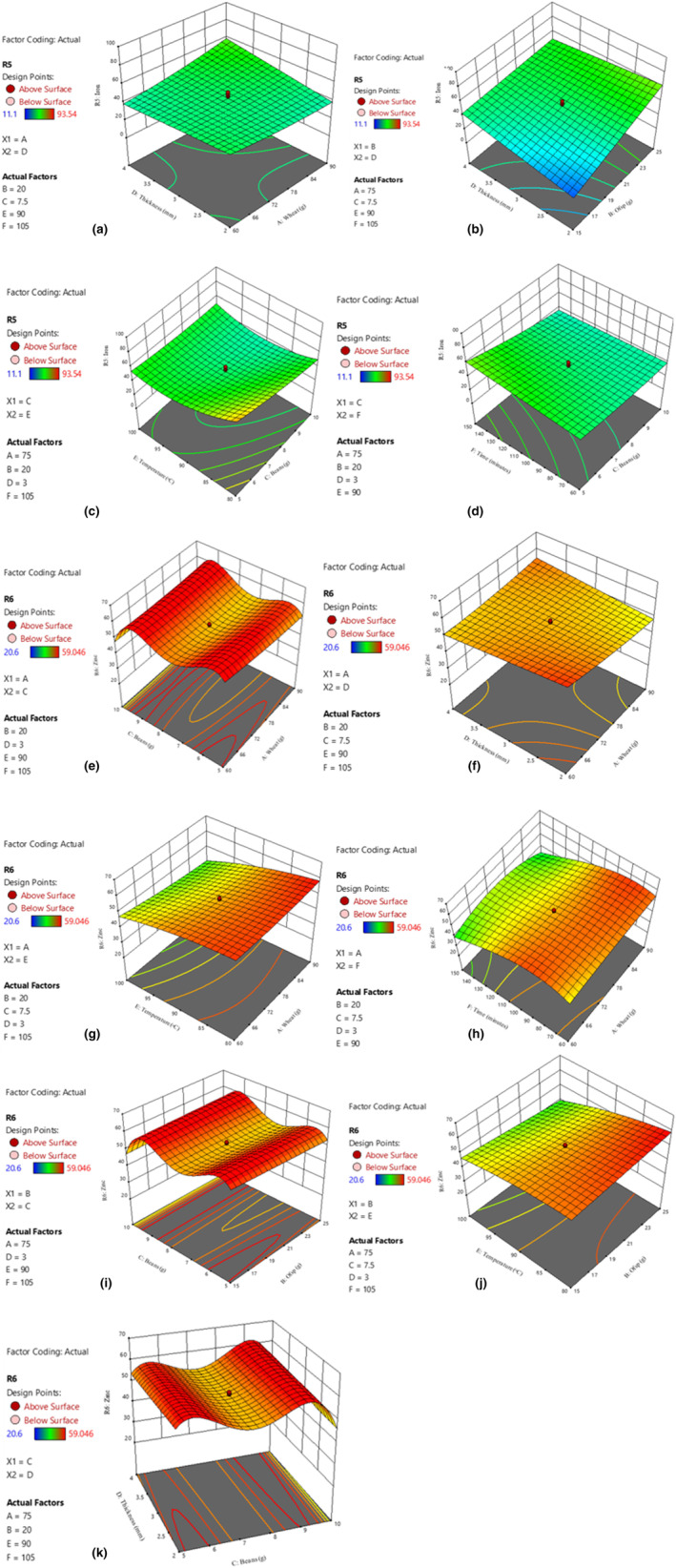
Effect of wheat and dough thickness (a); and OFSP and dough thickness (b); beans and drying temperature (c); and beans and drying time (d) on iron content; effect of wheat and beans (e); and wheat and dough thickness (f); wheat and drying temperature (g); and wheat and drying time (h); OFSP and beans (i); and OFSP and drying temperature (j); beans and dough thickness (k) on zinc content of enriched noodles.

The iron content of nutritionally enhanced noodles ranged between 11.1 and 84.9 ppm while that of zinc content ranged between 20.6 and 59.1 ppm (Table 2). Figure 5a–k show the relationship between independent variables (wheat, OFSP, beans, dough thickness, drying temperature, and drying time) and minerals (iron and zinc) content. The experimental and theoretical results were in close agreement, as indicated by the proximity between *R* ^2^ (0.86) and adjusted *R* ^2^ (0.72) values for iron and *R* ^2^ (0.96) and adjusted *R* ^2^ (0.91) values for zinc (Table 3). The relationship between minerals’ content and independent variables (ingredients and processing parameters) is given in Equations 6 and 7 for iron and zinc, respectively. The linear terms for wheat, OFSP, and beans had a significant effect on iron (*p* < .05). Whereas all the linear terms for wheat, OFSP, and beans had no significant effect on zinc (*p* > .05).

The high iron and zinc in nutritionally enhanced noodles are attributable to the biofortified bean powder and orange‐fleshed sweet potatoes added as ingredients in the production of the noodles. The higher the bean powder and orange‐fleshed sweet potato used in the formulation, the higher the mineral content of iron and zinc (Pakhare et al., 2018). This is because, biofortified bean varieties have a high content of key nutrients, including iron and zinc (Beebe, 2020). Additionally, orange‐fleshed sweet potatoes are naturally rich in several nutrients, including vitamin C, which can enhance the absorption of nonheme iron (the type of iron found in plant‐based foods) and zinc (Neela & Fanta, 2019).

The results of this study show that partial substitution of wheat with biofortified beans and OFSP produced noodles with higher nutrient content and nutrient bioavailability (Whittaker, 1998). This has the potential to contribute to improved nutrition, especially in regions where micronutrient deficiencies are a concern.

### 3.3 Optimization

The numerical multiple response optimization was performed to determine the optimal combination of process and product parameters to produce the most desirable OFSP‐based functional noodles with the most desired moisture content, hardness, protein, dietary fiber, iron, and zinc content. During optimization, the importance of the response variables (moisture content, hardness, protein, dietary fiber, iron, and zinc content) was set at 3 for all variables. Optimization was applied to the selected ranges of independent variables (the content of wheat flour, orange‐fleshed sweet potatoes, beans powder, dough thickness, drying temperature, and drying time). A combination of optimum levels of the different independent variables (the content of wheat flour, OFSP, bean, dough thickness, drying temperature, and drying time) with predicted values for the response variables (moisture, hardness, protein, dietary fiber, iron, and zinc) generated by the software is shown in Table 4.

**TABLE 4:** Optimized process protocol for production of OFSP‐based noodles from Design‐Expert software.

No.	Wheat (g)	OFSP (g)	Beans (g)	Dough thickness (mm)	Drying temperature (°C)	Drying time (min)	Moisture (%)	Hardness (*N*)	Protein (%)	Dietary fiber (%)	Iron (ppm)	Zinc (ppm)	Desirability	Comment
1	83.0	24.6	6.2	2.0	80.0	143.4	5.9	11.0	34.5	11.9	86.9	50.5	0.819	Selected
2	84.4	16.5	5.4	3.8	80.0	150.0	6.1	13.0	34.5	11.5	92.3	49.7	0.818	
3	85.5	24.3	9.3	2.0	80.0	143.8	3.0	8.3	39.7	13.5	62.0	59.1	0.817	
4	83.7	24.8	6.1	2.0	80.0	140.3	6.9	10.5	34.5	11.6	87.0	51.7	0.816	
5	82.6	16.1	5.4	3.6	80.0	150.0	7.4	12.1	34.5	11.6	87.3	51.8	0.815	
6	83.3	16.5	5.4	3.8	80.0	150.0	6.7	13.0	36.5	11.4	91.4	50.1	0.815	
7	88.8	24.3	8.9	2.6	80.0	142.9	6.1	10.9	34.5	14.4	58.4	58.4	0.815	
8	84.3	25.0	6.0	2.0	80.0	138.7	7.2	10.3	34.5	11.5	86.8	52.1	0.814	

The combination with the highest desirability value of 0.819 was selected. This corresponded to a formulation consisting of 73.0%, 21.5%, and 5.5% of wheat, orange‐fleshed sweet potatoes, and beans, respectively, and processing conditions of 2.0 mm thickness, 80.0°C (drying temperature), and 143.4 min (drying time). The predicted values for response variables for noodles processed under these optimum conditions were 5.9% moisture content, 11.0 N hardness, 34.5% protein, 11.9% dietary fiber, 86.9 ppm iron, and 50.53 ppm zinc. There was no significant difference between the observed values and those predicted by the Design‐Expert software, as shown in Table 5. This shows that the model adequately predicted the responses.

**TABLE 5:** Results obtained in the validation of the selected optimized process protocol for OFSP‐based noodles.

	Moisture (%)	Hardness (*N*)	Protein (%)	Dietary fiber (%)	Iron (ppm)	Zinc (ppm)
Observed values	5.98^a^ ± 0.07^b^	11.14^a^ ± 0.93^b^	35.06^a^ ± 0.39^b^	11.77^a^ ± 0.67^b^	83.24^a^ ± 7.08^b^	52.12^a^ ± 1.00^b^
Optimum predicted model	5.9^a^	11^a^	34.5^a^	11.9^a^	86.9^a^	50.5^a^
Deviation^c^	0.0134	0.0126	0.0160	0.0110	0.0441	0.0311

^a^ No significant difference between the observed values and the predicted values.

^b^ Presented data are at 95% confidence interval.

^**c**^ Deviation = (observed – predicted)/observed.

### 3.4 Sensory acceptability

The mean sensory scores for the noodles tested ranged between 7.4 (liked moderately) and 8.0 (liked very much) assessed (Table 6). There was no significant difference between the sensory scores for the control (plain wheat noodles) and the nutrient‐enriched noodles developed. In some cases, enrichment of noodles with other ingredients has been shown to negatively affect sensory attributes. A study by Cumhur et al. (2022) showed that the enrichment of noodles with okra seed powder negatively affected the color, taste, flavor, odor, appearance, and overall acceptability of the final product. The sensory evaluation results of the current study were in contrast to the findings by Cumhur et al. (2022) since the substitution of wheat flour with orange‐fleshed sweet potatoes and biofortified beans resulted in a product that was as acceptable by sensory panelists as plain wheat noodles. Similar findings have been reported by Kindeya et al. (2021) when the overall acceptability of biscuits was improved by incorporating OFSP powder and haricot bean flour.

**TABLE 6:** Sensory evaluation of OFSP‐based noodles.

Attributes	Appearance	Color	Aroma	Taste	Mouthfeel	Aftertaste	Overall acceptability
Control	7.7^a^ ± 0.99	7.7^a^ ± 1.18	7.8^a^ ± 1.17	7.6^a^ ± 1.17	7.5^a^ ± 1.22	7.6^a^ ± 1.11	8.0^a^ ± 0.95
OFSP‐based noodles	7.44^a^ ± 1.18	7.6^a^ ± 1.14	7.9^a^ ± 1.10	7.5^a^ ± 1.27	7.6^a^ ± 1.09	7.6^a^ ± 1.12	7.8^a^ ± 1.06

*Note*: Values are means ± standard deviation (*n* = 50). Means in each column with different superscripts are significantly different (*p* ≤ .05). Scores for each sensory attribute based on a 9‐point hedonic scale: 1 = disliked extremely, 2 = disliked very much, 3 = disliked moderately, 4 = disliked slightly, 5 = neither liked nor disliked, 6 = liked slightly, 7 = liked moderately, 8 = liked very much, and 9 = liked extremely.

## 4 CONCLUSION

This study aimed to develop an optimized formulation and processing protocol for noodles enriched with orange‐fleshed sweet potato and biofortified beans and to evaluate the sensory acceptability of the developed noodles. By applying the response surface methodology, an optimal protocol for the production of functional noodles was developed. This corresponded to a formulation consisting of 73.0%, 21.5%, and 5.5% of wheat, orange‐fleshed sweet potatoes, and beans, respectively, and processing conditions of 2.0 mm thickness, 80°C (drying temperature), and 143.4 min (drying time). The predicted values for response variables for noodles processed under these optimum conditions were 5.9% moisture content, 11.0 *N* hardness, 34.5% protein, 11.9% dietary fiber, 86.9 ppm iron, and 50.53 ppm zinc. The developed noodles were highly acceptable with an overall acceptability score of 7.8 on the 9‐point hedonic scale. Partial substitution of wheat flour with OFSP and biofortified bean powder did not affect the sensory acceptance of the developed noodles. These findings confirmed the potential for OFSP and beans as ingredients in the production of noodles. This creates a potential market for local foods like sweet potatoes and beans through value addition, with prospects to benefit farmers and other value chain actors.

## AUTHOR CONTRIBUTIONS

**Janet Natocho:** Conceptualization (equal); data curation (equal); formal analysis (equal); investigation (equal); validation (equal); writing – original draft (equal); writing – review and editing (equal). **Robert Mugabi:** Conceptualization (equal); formal analysis (equal); methodology (equal); supervision (equal); writing – original draft (equal); writing – review and editing (equal). **John H. Muyonga:** Conceptualization (equal); funding acquisition (equal); investigation (equal); methodology (equal); project administration (equal); resources (equal); supervision (equal); writing – original draft (equal); writing – review and editing (equal).

## FUNDING INFORMATION

The authors would like to extend appreciation to the European Union (EU) for funding through the oodLAND (Food and Local, Agricultural, and Nutrition Diversity) project, Makerere University (Grant agreement number: 862802) that enabled the successful execution of this research.

## CONFLICT OF INTEREST STATEMENT

The authors declare no conflict of interest.

## Data Availability

The data used to support the findings of this study are included in the article. Should further data or information be required, these are available upon reasonable request from the corresponding author.
